# Oxidative Stress and Antioxidants in Neurodegenerative Disorders

**DOI:** 10.3390/antiox12020517

**Published:** 2023-02-18

**Authors:** Edward O. Olufunmilayo, Michelle B. Gerke-Duncan, R. M. Damian Holsinger

**Affiliations:** 1 Laboratory of Molecular Neuroscience and Dementia, School of Medical Sciences, Faculty of Medicine and Health, The University of Sydney, Camperdown, NSW 2050, Australia; 2 Department of Medicine, University College Hospital, Queen Elizabeth Road, Oritamefa, Ibadan 5116, PMB, Nigeria; 3 Education Innovation, School of Medical Sciences, Faculty of Medicine and Health, The University of Sydney, Sydney, NSW 2006, Australia; 4 Neuroscience, School of Medical Sciences, Faculty of Medicine and Health, The University of Sydney, Sydney, NSW 2006, Australia

**Keywords:** Alzheimer’s disease, Parkinson’s disease, Huntington’s disease, amyotrophic lateral sclerosis, mitochondrial stress, reactive oxygen species, reactive nitrogen species, homeostasis, free radicals

## Abstract

Neurodegenerative disorders constitute a substantial proportion of neurological diseases with significant public health importance. The pathophysiology of neurodegenerative diseases is characterized by a complex interplay of various general and disease-specific factors that lead to the end point of neuronal degeneration and loss, and the eventual clinical manifestations. Oxidative stress is the result of an imbalance between pro-oxidant species and antioxidant systems, characterized by an elevation in the levels of reactive oxygen and reactive nitrogen species, and a reduction in the levels of endogenous antioxidants. Recent studies have increasingly highlighted oxidative stress and associated mitochondrial dysfunction to be important players in the pathophysiologic processes involved in neurodegenerative conditions. In this article, we review the current knowledge of the general effects of oxidative stress on the central nervous system, the different specific routes by which oxidative stress influences the pathophysiologic processes involved in Alzheimer’s disease, Parkinson’s disease, Amyotrophic Lateral Sclerosis and Huntington’s disease, and how oxidative stress may be therapeutically reversed/mitigated in order to stall the pathological progression of these neurodegenerative disorders to bring about clinical benefits.

## 1. Introduction

Oxidative stress is a phenomenon resulting from an imbalance between the production and accumulation of reactive oxygen species (ROS) and reactive nitrogen species (RNS) in cells and tissues and the ability of cellular machineries to eliminate these by-products. It results from perturbations in homeostatic mechanisms involved in pro- and antioxidant balance. ROS/RNS are by-products of cellular respiration, the process during which life-sustaining cellular reactions occur. Oxidative stress is a common denominator of neuronal cell loss associated with neurodegenerative disorders including Alzheimer’s disease (AD), Parkinson’s disease (PD), Amyotrophic Lateral Sclerosis (ALS) and Huntington’s disease (HD) [1], as well as brain and spinal cord damage following stroke [2] and traumatic brain injury (TBI) [3]. The accumulation of both ROS and RNS has also been found to be involved in mitochondrial dysfunction, leading to deficits in energy production, changes in metal homeostasis and the accumulation of toxic protein aggregates that typify many neurodegenerative disorders. These eventuate in the activation of cell death pathways via apoptosis, necrosis, ferroptosis or autophagy.

The key physiological roles of ROS/RNS include the regulation of cellular homeostasis via the mediation of processes including redox signaling, defense against pathogens and protein folding [4]. Many cellular organelles have an intrinsic ability to scavenge and ‘mop up’ ROS and RNS [5]. Generally, an antioxidant defense system based predominantly on the enzymes catalase (CAT), superoxide dismutase (SOD), glutathione peroxidase (GPx) and glutathione reductase (GSR) protects the body and its tissues from ROS/RNS-induced cellular damage [6]. However, when reactive species reach critical concentrations within cells, their deleterious effects rapidly overshadow their benefits as antioxidant countermeasures are overwhelmed. The inability to maintain redox homeostasis, either due to an overproduction of or reduced/impaired elimination of these reactive species, leads to oxidative stress. The presence of oxidative stress leads to the perturbation of many cellular functions via the interaction of reactive species with cellular components such as DNA, RNA, amino acids, carbohydrates, lipids and proteins. Such damage to cells, especially neurons, has been shown to be highly detrimental to their survival [7]. As such, oxidative stress has been shown to contribute to the aetiology of various metabolic disorders [8] and malignant conditions [9], as well as neurodegenerative diseases [10].

Given the established important roles that oxidative stress plays in neuronal cell damage and death, it is prudent to assume that reinforcing the endogenous antioxidant systems and mechanisms may play a crucial protective role against the development of various neurodegenerative disorders. A large volume of recent research efforts has been directed towards developing antioxidant-based approaches to challenge oxidative stress-induced neuronal dysfunction and loss that is a typical feature of various neurological diseases. Therefore, in this review, we will examine the current knowledge of the widespread effects of oxidative stress on the central nervous system, how these effects contribute to the development of a number of neurodegenerative disorders and various antioxidant-based therapeutic strategies that have been developed to address these neurodegenerative conditions.

## 2. Oxidative Stress and Pro-/Antioxidant Balance

The chemical reactive potential of the oxygen molecule is based on its atomic structure, possessing two unpaired electrons in its outer shell. This property implies that oxygen oxidizes other substances by readily accepting electrons via partial or complete transfer, itself being reduced in the process. This promotes reactions that form the basis for important processes such as oxidative burst activity in macrophages, respiration (where oxygen is reduced in four single electron reactions) and the microsomal electron transport chain (ETC) (via the cytochrome system) [11]. Biological oxidants that possess the potential to cause oxidative damage are by-products of endogenous and exogenous reactions that involve oxygen and nitrogen as chemical components.

As has been mentioned, ROS/RNS play important regulatory and mediator functions in cellular metabolism at physiological concentrations. However, uncontrolled increases in the levels of these reactive species lead to a chain of reactions that generate free radicals as by-products and subsequently impair cellular function through the widespread damage of biomolecules. This, in part, is due to the high reactivity of ROS and RNS with cellular components including lipids, proteins, carbohydrates and nucleic acids. Hence, an appropriate antioxidant barrier is required to limit the concentration of these reactive species to a physiological level. Uncontrolled generation of ROS/RNS that exceeds maximum cellular capacity leads to a perturbed pro-antioxidant equilibrium and, eventually, to the development of a state of oxidative stress. In addition to endogenous processes that drive oxidative stress, exogenous processes and factors such as xenobiotics, ionizing radiation, viral and bacterial infections, poor diet, alcohol consumption and smoking, amongst others, also contribute to the generation of reactive species [12].

The best-known ROS and RNS are singlet oxygen (O_2_), superoxide anion radicals (O_2_^•−^), hydroxyl radicals (HO^•^), hydrogen peroxide (H_2_O_2_), nitric oxide (NO) and peroxynitrite anions (ONOO^−^) [13,14]. Mitochondria are the single most important cellular organelles involved in the generation of ROS and RNS [15], mostly as a consequence (by-products) of aerobic respiratory processes, which are mediated by protein complexes within the inner mitochondrial membrane. Being high-energy-consuming cells with large numbers of mitochondria, neurons are more likely to produce higher amounts of these reactive species, with a consequent significant risk of cellular damage.

### Generation of Reactive Species

**Figure 1:**
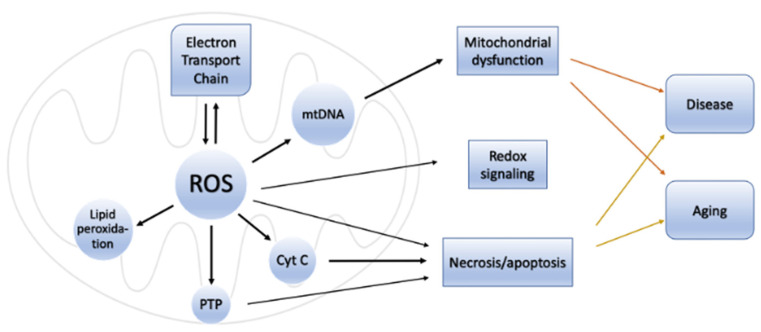
Effects of ROS production by mitochondria. The generation of ROS as a by-product of cellular respiration via the electron transport chain in mitochondria can have deleterious effects in the cell and tissue involved. ROS can cause oxidative damage to mitochondrial DNA and protein, resulting in mutations and mitochondrial dysfunction that impair the ability of mitochondria to synthesize ATP, leading to disease. Reactive species can also lead to the induction of a permeability transition pore (PTP) in mitochondria, rendering the inner membrane of the mitochondria more permeable to small molecules during episodes of ischemia/reperfusion injury. ROS can also lead to the oxidative degradation of lipid molecules (lipid peroxidation), especially within the cell membrane, resulting in cell damage and death. Reactive species can also lead to the release of intermembrane proteins including cytochrome c (Cyt C) into the cytosol of the cell, which, in turn, can activate apoptotic machinery within a cell, leading to cell death.

The primary reactive species produced within the mitochondria is superoxide. This molecule is subsequently converted to hydrogen peroxide by the action of the enzyme superoxide dismutase (SOD). These events take place within four membrane-bound, multi-subunit protein complexes referred to as the respiratory chain or electron transport chain (ETC) located within the inner mitochondrial membrane. The four proteins consist of complex I (a reduced form of nicotinamide adenine dinucleotide (NADH) ubiquinone reductase), complex II (succinate ubiquinone reductase), complex III (ubiquinol cytochrome c reductase) and complex IV (cytochrome c oxidase) [1]. Complexes I–IV accept and then transfer electrons between themselves and specific electron carriers within the inner mitochondrial membrane in a sequential manner in order to pump protons into the intermembrane space of the mitochondria, producing an electrochemical gradient needed for the production of ATP by ATP synthase (also referred to as complex V), via oxidative phosphorylation [16,17]. Complex IV transfers electrons to oxygen, which undergoes a four-electron reduction, leading to the generation of water molecules. Single electrons may, however, leak during the processes involved in electron transport, especially from the electron carriers (ubiquinone and cytochrome c) involved in the ETC. When this happens, the electrons react with molecular oxygen to form superoxide (O_2_^•−^) and, later, H_2_O_2_ and HO^•^ [18]. These species can be detrimental to cells and contribute to aging and neurodegenerative changes when affecting cells of the nervous system (Figure 1).

The superoxide radical (O_2_^•−^) that is produced via the enzymatic processes by NADPH oxidase, xanthine oxidase and peroxidases subsequently undergoes several reactions that eventuate in the production of more free radicals including H_2_O_2_, hydroxyl radicals, peroxynitrite and hypochloric acid (HOCl), amongst others (Table 1). H_2_O_2_ (a nonradical ROS) is also produced by multiple oxidases, including amino acid oxidase and xanthine oxidase [19]. The hydroxyl radical (OH^•^) is regarded the most reactive free radical species in vivo, and it is often produced from H_2_O_2_ in the presence of a metallic ion, typically Fe^2+^, which plays a catalytic role in a process referred to as the Fenton reaction [20,21,22].

In mitochondria, nitric oxide is produced from L-arginine via nitric oxide synthase (NOS) (Table 2). The enzyme consists of three isoforms, which are differentially distributed across different tissues. Whilst Ca^2+^-dependent neuronal NOS (nNOS) is expressed in macrophages, microglia and astrocytes, the vascular endothelium expresses endothelial NOS (eNOS) as well as Ca^2+^-independent inducible NOS (iNOS) [23]. Numerous critical functions of the central nervous system rely on NO, including the regulation of cerebral blood flow, memory and learning processes, amongst others [24]. In addition, iNOS-generated NO is produced in response to the presence of pathogen-associated molecular patterns (PAMPs), small, microbial-associated molecular motifs that play a role in the modulation of cytokine production by the immune system [25]. Nitric oxide rapidly reacts with superoxide to form peroxynitrite. RNSs also contribute to oxidative stress. Peroxynitrite rapidly decomposes into hydroxyl radicals, nitrogen dioxide radicals (NO_2_^•^) and nitryl cations (NO_2_^+^). Nitrogen dioxide radicals are also produced from the auto-oxidation of nitric oxide species, whilst nitryl cations (also referred to as nitronium ions) are produced from the protonation of nitric acid and oxidation (via electron removal) of the nitrogen dioxide molecule [26,27]. All of these reactive species can cause oxidative damage to neurons [28]. Free radicals are also often produced by various nonenzymatic processes outside the mitochondria, an important example being the reaction of oxygen molecules with organic compounds that are triggered when cells are exposed to ionizing radiation [19].

Excessive levels of hydroxyl and peroxynitrite radicals cause the activation of lipid peroxidation that results in widespread damage to cellular and organelle membranes and lipoproteins, with acrolein, isoprostanes, malondialdehyde (MDA), 4-hydroxynonenal (HNE) and conjugated dienes formed as by-products of this reaction [12]. Constituting a radical chain reaction, lipid peroxidation spreads rapidly, affecting a substantial number of lipid molecules [29]. The CNS has a particularly high lipid content [30], resulting from the morphology of neurons and their cellular projections, including axons that are ensheathed by myelin. This high lipid content translates into a higher load of toxic aldehyde products of lipid peroxidation reacting with DNA and protein molecules, altering their structures and, consequently, their functions [31]. Proteins are also highly susceptible to damage caused by oxidative stress, as they cause conformational alterations that may either impair protein function or result in a total loss of their functional capabilities [32]. The nonenzymatic reactions of ROS and RNS with cellular carbohydrates result in the formation of advanced glycation end-products (AGEs), which contribute to the development of neurodegenerative diseases [33]. DNA is also highly susceptible to oxidative stress-induced damage, often characterized by the formation of 8-oxo-2′-deoxyguanosine (8-OHdG) that results from damage to guanine nucleotides [34]. Damage to DNA has far-reaching consequences for cellular metabolism, including the impairment of protein synthesis and potentially deleterious mutagenesis. Levels of 8-OHdG and a range of other altered biological compounds are being widely proposed and adopted as tissue biomarkers of oxidative stress [35,36]. In addition, there are numerous other tissue-specific and nonspecific biomarkers that signify the presence of ROS- and RNS-induced damage in cells and tissue [37,38,39].

If not controlled within strict limits, oxidative stress can result in substantial neuronal damage and may herald the pathogenesis of numerous neurodegenerative diseases. Large bodies of evidence have demonstrated that increased neuronal ROS/RNS levels and the accompanying oxidative damage correlate with the extent of neurodegeneration in neurodegenerative disorders. This underscores the requirement of a functional cellular antioxidant system that will counter the deleterious effects of excessive ROS/RNS, maintain pro-/antioxidant balance and possibly prevent or delay the onset of neurodegenerative conditions.

We will next discuss the general effects of oxidative stress on the CNS, followed by a more detailed description of its effects in four common neurodegenerative diseases. The final section of this review will focus on key antioxidants and their potential as therapeutics for these diseases.

## 3. General Effects of Oxidative Stress on the Central Nervous System

The CNS has a high metabolic rate that necessitates extensive energy production by mitochondria. This high metabolic rate, required for the production of ATP via the electron transport chain and oxidative phosphorylation, implies that neurons and glia (microglia and astrocytes) are more likely to generate large quantities of ROS/RNS through the mechanisms discussed above. Furthermore, neurons and other CNS cells are particularly vulnerable to oxidative stress due to their intrinsic properties. The biochemical, cellular and tissue impacts of oxidative stress vary depending on the impacted region of the CNS. The cumulative effects of extended periods of oxidative stress manifest in the form of a vast number of CNS disorders, including neurodegenerative conditions.

The hippocampus, amygdala, prefrontal cortex and cerebellar granular cells are regarded as the structures of the brain most vulnerable to oxidative stress [40]. Some of the most important explanations for this selective vulnerability to oxidative stress include significantly higher ATP requirements in these cells compared to others, which renders them exquisitely sensitive to stressors such as differential signaling requirements for ROS/RNS, differences in intrinsic oxidative stress levels among different neuronal populations, differences in glial–neuronal crosstalk mechanisms, differences in intrinsic DNA repair abilities and differences in calcium signaling processes among different neuronal populations [40]. Consequently, these structures are amongst the earliest to undergo functional decline in states of oxidative stress. The findings that the aforementioned CNS structures are also the most severely affected in a number of neurodegenerative diseases, including Alzheimer’s disease [41], supports the notion that oxidative stress may represent a “trigger” that makes nervous system cells vulnerable to a cascade of toxic insults that ensue. The hippocampus is critical for various aspects of learning and memory [42], whilst the ventromedial prefrontal cortex (PFC) acts to integrate various aspects of memory into neocortical regions [43,44]. Supporting these observations, high levels of oxidative stress have been shown to correlate strongly with behavioral deficits and impairments in temporal and spatial memory, learning and retention in animal models of aging [45,46].

During oxidative stress, the hippocampus undergoes significant biochemical changes that eventually affect neuronal connections and function. The cornu ammonis (CA)3 neurons of the hippocampus exhibit structural plasticity with significant regenerative/remodeling capacity [47,48]. Numerous studies have demonstrated the vulnerability of the pyramidal cells of CA3 and granule cells of the dentate gyrus (DG) to oxidative stress, whilst others have shown the vulnerability of pyramidal cells of area CA1 to oxidative damage [49,50,51,52]. Regardless of which areas of the hippocampus are affected, there are significant functional consequences of oxidative stress on the hippocampus and dentate gyrus. During chronic stress, including those mediated by ROS/RNS, neurons within the amygdala and prefrontal cortex have been shown to undergo dendritic alterations characterized by dendritic contraction in the medial PFC and dendritic growth in neurons within the amygdala, with resultant perturbations in neuronal connectivity [53,54,55,56]. These studies demonstrate that oxidative stress compromises the biochemical and structural integrity of the hippocampus, the amygdala and prefrontal cortex, areas often central to the pathology of a number of neurodegenerative conditions.

In vitro models have been used extensively to study the effects of oxidative stress. The mouse neuroblastoma-derived HT4 cell line treated with cadmium (which induces oxidative stress) revealed increased cellular protein ubiquitination and subsequent vast activation of the ubiquitin–proteasome machinery, leading to the degradation of the ubiquitinated proteins [57]. This result suggests that oxidative stress may result in an overwhelming of the ubiquitin–peroxisome system, with a resultant accumulation of some of these altered proteins within the neurons. These protein deposits may then perturb normal neuronal function, induce a state of endoplasmic reticulum (ER) stress, as depicted by an increase in levels of GRP78 (78-kDa glucose-regulated protein), a chaperone protein involved in the unfolded protein response (UPR) that eventuates in the activation of the autophagic pathway [58]. Uncontrolled and unchecked accumulation of altered and misfolded proteins in the ER and cytosol is central to the pathophysiology of many pathologies, including neurodegenerative disorders [58,59].

Apart from neurons, oxidative stress has also been shown to adversely affect other cellular components of the CNS. Astrocytes are known to contribute to appropriate inflammatory responses and innate immunity within the CNS. Oxidative stress causes astrocyte-associated inflammation and concomitant astrogliosis [60,61]. Reactive astrogliosis is a cardinal feature of a wide range of CNS disorders, including neurodegenerative diseases [62]. ROS and RNS have been shown to activate a number of inflammatory signaling pathways in astrocytes, resulting in the release of inflammatory mediators, often perpetuating astrogliosis [63]. ROS can activate the astrocytic NLRP3 ((NOD-, LRR- and pyrin domain-containing protein 3) inflammatory cascade by activating procaspase-1. The resulting caspase-1 mediates the release of the proinflammatory cytokines, IL-1β and IL-18 [64,65]. Excessive release of these inflammatory mediators exacerbates neuronal damage. H_2_O_2_ in particular has been shown to cause ROS-induced cell death in astrocytes by upregulating NF-κB activity [66]. Since astrocytes and microglia are in a constant state of crosstalk [67], it can be inferred that the proinflammatory cytokines released by astrocytes in response to oxidative stress eventually activate microglia, thereby propagating neuroinflammation [68,69]. In addition, the established bidirectional relationship between microglia and astrocytes implies that microglia also activate astrocytes through cytokine-mediated crosstalk [66] to promote astrogliosis and the propagation of neurodegeneration, as activated microglia are known to play many crucial roles in both the progression and mitigation of the pathophysiologic processes involved in many neurodegenerative diseases [5,70]. Oligodendrocytes are also susceptible to oxidative stress. Studies have shown that oxidative stress is harmful to oligodendrocytes, and the ensuing death of these cells may result in widespread demyelination, a common feature of a number of neurological diseases including neurodegenerative conditions [71,72,73].

The studies listed above are but a small sample of a large volume of studies that suggest that oxidative stress and concomitant inflammation are significant factors in promoting direct neuronal damage, astrogliosis, glial scar formation and demyelination that contribute to the development of neurodegenerative diseases.

## 4. Roles of Oxidative Stress in Specific Neurodegenerative Conditions

We have discussed how oxidative stress exerts adverse effects on different areas and cellular components of the CNS, and how these may generally contribute to the pathophysiology of neurodegenerative conditions as a collective entity. We will next highlight the specific contributions of oxidative stress to the pathophysiologic mechanisms involved in specific neurodegenerative diseases, with a focus on Alzheimer’s disease, Parkinson’s disease, Amyotrophic Lateral Sclerosis and Huntington’s disease.

### 4.1. Alzheimer’s Disease

Alzheimer’s disease (AD) is the most common neurodegenerative disorder and a leading cause of dementia. The disease is pathologically characterized by the extracellular deposition of amyloid-β protein (Aβ) in the form of neuritic plaques and the intracellular accumulation of the hyperphosphorylated cytoskeletal protein tau into neurofibrillary tangles [74]. These deposits eventually lead to neuronal dysfunction, synaptic loss and neuronal death. The disease presents clinically with progressive cognitive decline involving memory and higher executive function [75]. Many studies have shown that oxidative stress is, in fact, an important player in the pathogenesis of AD [76].

The roles of mitochondria in ROS/RNS generation have been discussed above. Accumulating evidence suggests that the process of aging is often accompanied by mitochondrial dysfunction, typically characterized by a reduction in the α subunit of the F1 component of ATP synthase, resulting in impaired ATP generation and the increased production of free radicals, with associated consequences [77,78]. These studies also reveal that dysfunctional mitochondria are important contributors to the pathogenesis of Alzheimer’s disease, as one of the early steps of AD is increased mitochondrial DNA oxidation, presumably resulting from increased free radical levels associated with aging. Aging-related decline in mitochondrial function has been reported to affect the expression and processing of the amyloid precursor protein (APP), producing Aβ oligomers that accumulate to form plaques in AD [79,80]. Aβ is a 40–42 amino acid peptide that is formed by the sequential cleavage of APP by BACE1 and the γ-secretase complex of proteins. The hydrophobic amino acid 25–35 region of Aβ stimulates the generation of reactive oxygen species, causing neuronal toxicity and stimulating a vicious cycle of amyloid generation [81,82,83]. Mark and colleagues [84] reported that incubating neurons with Aβ_42_ oligomers results in lipid peroxidation, as indicated by elevated levels of protein-bound 4-hydroxy-2-*trans*-nonenal (HNE). Other studies have reported similar findings of elevated HNE and isoprostanes in AD [85,86]. Smith et al. [87] also reported elevated 3-nitrotyrosine levels in AD models, indicating the role of widespread peroxynitrite-induced damage in the disease process. Elevated levels of 8-hydroxydeoxyguanosine (8-OHDG) in AD reflect widespread oxidative damage to both mitochondrial and nuclear DNA [88], and even RNA, potentially affecting the protein-synthesizing machinery [89]. Aβ_42_ oligomers, being largely hydrophobic, reside in the lipid bilayer of the cell and organelle membranes, and covalent bonding of the generated HNE to neuronal proteins leads to synaptic dysfunction and neuronal death [90].

Oxidative stress triggered by ROS/RNS in AD affects neuronal glucose and glutamate transport and mitochondrial membrane potential [91,92]. It also reduces the activity of the sodium–potassium ATPase (the pump that facilitates action potential generation) and impairs neuronal calcium homeostasis [91,92], factors that contribute to neuronal dysfunction and damage. The potential of Aβ to induce oxidative stress is most likely associated with the complexes it forms with redox active metals. The binding of iron, zinc and copper to Aβ has been established in promoting its aggregation into plaques. Of these metals, copper has been found to form the most stable complexes with Aβ, eventually generating superoxide and hydrogen peroxide [80]. The resulting oxidative stress caused by these metal–amyloid complexes has been demonstrated to contribute to excitotoxicity, whereby it promotes membrane depolarization and impairs mitochondrial function [93]. Mitochondrial dysfunction, as discussed earlier, promotes oxidative stress, thus propagating a vicious cycle. The accumulation of Aβ lowers the respiratory control ratio (RCR) as well as the production of ATP and increases the generation of ROS in HEK293 cells [94], supporting the presence of a positive feedback loop between ROA and Aβ. These free radicals have been shown to facilitate the downstream activation of cPLA2 (calcium-dependent phospholipase A2), resulting in membrane disturbances, arachidonic acid release and the activation of kinases [95], all of which are known to be important to the disease process. Nanetti et al. [96] also showed that lipoproteins isolated from AD patients can facilitate the production of nitric oxide via the upregulation of nitric oxide synthase and increased peroxynitrite production in astrocytes, thus reinforcing the role of other cellular components of the CNS in the interplay between oxidative stress and AD pathophysiology.

These studies point toward a bidirectional, reciprocal relationship between the progression of AD and oxidative stress, with each entity reinforcing the other.

### 4.2. Parkinson’s Disease

Central to the pathophysiology of Parkinson’s disease (PD) is the loss of dopaminergic neurons in substantia nigra pars compacta (SNpc), which is responsible for the characteristic motor symptoms associated with PD and forms the basis of many of the existing pharmacological therapies for the disease [97]. Mitochondrial dysfunction and oxidative stress have been consistently associated with the cascade of events leading to dopaminergic neuron degeneration [98,99]. Postmortem studies of PD brains consistently reveal elevated levels of markers of oxidative stress including 4-hydroxyl-2-nonenal (HNE) [100] and 8-hydroxy-deoxyguanosine and 8-hydroxy-guanosine [101]. In addition, toxins such as 1-methyl-4-phenyl-1,2,3,6-tetrahydropyridine (MPTP), 1,1′dimethyl-4,4′-bipyridinium dichloride (paraquat), rotenone and 6-hydroxydopamine (6-OHDA), known to cause Parkinson’s in humans and often used to induce Parkinson’s pathology in animal models, are all known to induce oxidative stress, thus establishing a definite role for oxidative damage in the disease process [102,103].

Many studies have either theorized or proven that factors including dopamine metabolism, high levels of iron and low glutathione (GSH) in SNpc are important instigators of oxidative stress with consequent loss of dopaminergic neurons in the PD brain. Under physiological conditions, levels of dopamine are regulated via oxidative metabolism by MAO-A (Monoamine oxidase-A) within catecholaminergic neurons. However, during the process of aging and in PD, MAO-B, localized in glial cells, takes over this function [104,105], resulting in the production of 3,4-dihydroxyphenyl-acetaldehyde and H_2_O_2_. The generated H_2_O_2_ permeates into nearby dopaminergic neurons where it undergoes a Fenton reaction, catalyzed by Fe^2+^, to form the highly reactive hydroxyl radical [106]. Mallajosyula and colleagues [107] showed that inducing MAO-B expression in astrocytes in adult transgenic mice results in selective and progressive loss of dopamine-producing neurons in SNpc.

Dopamine also undergoes auto-oxidation that is catalyzed by metals or enzymes such as tyrosinase, resulting in the formation of dopamine quinones [108], which cyclize to form aminochrome, a highly reactive compound capable of generating superoxide and depleting cellular NADPH stores [109]. NADPH is essential for the generation of reduced glutathione (GSH) by glutathione reductase and for the activity of the thioredoxin antioxidant system, where both play important roles in protecting cells from oxidative stress [110]. Analyses of postmortem PD brain tissue consistently reveal decreased levels of glutathione relative to glutathione disulfide (GSSG) (GSH:GSSG ratio) in the substantia nigra, signifying a major role for oxidative stress resulting from the impairment of these major antioxidant systems in PD [111,112,113].

Iron is an essential cofactor for the normal function of many neuronal proteins [114]. Brain tissue from PD subjects has higher levels of iron in substantia nigra compared to age-matched normal controls [111,115]. Ferric (Fe^3+^) and ferrous (Fe^2+^) iron react with superoxide and hydrogen peroxide, respectively, to generate highly reactive hydroxyl radicals, which, combined with the products of dopamine oxidation, cause neurotoxicity [116,117]. Conversely, oxidative stress also increases cellular levels of iron via its release from the superoxide-induced liberation from ferritin, heme proteins including hemoglobin, cytochrome c via the activity of peroxidase and from iron–sulfur proteins by peroxynitrite [118,119].

The general roles of mitochondrial dysfunction in the generation of ROS/RNS and the sustenance of oxidative stress have been discussed in earlier sections of this review. Mitochondrial dysfunction was initially associated with PD when postmortem analysis of brain tissues from narcotic drug users with MPTP-induced parkinsonism showed significant dopaminergic neuron loss in substantia nigra [120]. The effects of MPTP were first discovered in the early 1980s when a batch of the illicit narcotic desmethylprodine or 1-methyl-4-phenyl-4-propionoxypiperidine (MPPP), an opioid analgesic, became contaminated to form MPTP. Users displayed profoundly disabling parkinsonism. MPTP is capable of crossing the blood–brain barrier, where it is sequestered by astrocytes and metabolized by MAO-B into 1-methyl-4-phenylpyridinuim (MPP^+^), which is then released into the extracellular environment. MPP^+^ is selectively taken up by dopaminergic neurons, where it inhibits complex I of the electron transport chain in mitochondria [121], with a number of studies showing reduced complex I activity and ubiquinone in the substantia nigra of these individuals [122,123]. The reduced activity of components of the electron transport chain, as discussed earlier, results in significant electron leak, with subsequent generation of free radicals that mediate neurotoxicity. Dopaminergic neurons from PD brains have also shown impairments in the production of mitochondrial proteins, further reflecting the state of mitochondrial dysfunction in PD [124].

A pathological hallmark of PD is the presence of Lewy bodies, which are neuronal inclusion bodies composed of abnormal fibrils of the protein alpha-synuclein [125]. Aggregates of alpha-synuclein have been associated with increased oxidative stress [126]. The presence of nitrosylated iron regulated protein 2 (IRP) in Lewy bodies in the substantia nigra of PD patients also reflects the roles of oxidative stress-induced iron dysregulation in the PD brain [127]. Alpha-synuclein can also induce oxidative stress by reducing the activity of complex I, thereby impairing mitochondrial function [128,129,130]. Complex relationships between oxidative stress and other PD-related proteins including Parkin [131], LRRK2 [132], DJ-1 [133] and PINK1 [134] also contribute to the disease process.

### 4.3. Amyotrophic Lateral Sclerosis (ALS)

Amyotrophic lateral sclerosis (ALS), also known as Lou Gehrig’s disease or Charcot disease, is the most common motor neuron disease. Characterized by the progressive loss of both upper and lower motor neurons within the brain stem and spinal cord, the disease causes muscle weakness, progressive muscle atrophy and paralysis, eventually leading to respiratory failure and death [135]. The average 10-year survival rate is 5–10%, and no effective treatment modality has yet been discovered. Current treatments for the disease include three drugs that have been approved by the US Food and Drug Administration: riluzole, a neuroprotective agent that extends life expectancy by approximately 3–6 months, the intravenous administration of edaravone, an antioxidant that slows the decline of motor function [136] and the recently approved sodium phenylbutyrate/taurursodiol, a drug shown to shown to prolong survival and slow functional decline in ALS patients [137]. A vast majority (90–95%) of patients with ALS have the sporadic form of the disease (sALS) where no single identifiable cause has been discovered. The remainder of patients have the heritable/familial (fALS) form of the disease that is associated with an earlier age of onset [135,138]. Some of the most prevalent genetic mutations in fALS are those involving chromosome 9 open reading frame 72 (C9orf72) [139], Cu^2+^/Zn^2+^ superoxide dismutase type-1 (SOD1) [140,141] TAR DNA-Binding Protein (TARDBP) [142] and fused-in sarcoma (FUS) [143]. The proposed molecular mechanisms underpinning neurodegeneration in ALS include altered RNA metabolism, endoplasmic reticulum stress, impaired protein folding, aggregation and trafficking, glutamate excitotoxicity, defective axonal transport, inflammation, mitochondrial dysfunction and oxidative stress [144].

Evidence of oxidative damage to DNA, proteins and lipids in postmortem ALS neuronal tissue [145,146] suggest the involvement of oxidative stress in ALS pathophysiology. There have also been reports of increased protein carbonyls, 8-hydroxy-2′-deoxyguanosine and malondialdehyde-modified proteins [146], 4-hydroxynonenal conjugates [147,148] and nitrotyrosine products [149] in spinal cord ALS tissue, all of which are important markers of oxidative stress. There have also been reports of reductions in antioxidant molecules including catalase, glutathione, glutathione reductase and glucose-6-phosphate dehydrogenase in the erythrocytes of ALS patients [150].

Mutations in SOD1 account for ~20% of fALS cases and are a well-studied cause of the disease, involving oxidative stress and mitochondrial dysfunction [151]. The enzyme is a Cu-Zn metalloprotein that catalyzes the conversion of superoxide radicals into H_2_O_2_ and O_2_, and is located within the nucleus, cytosol, peroxisomes and mitochondria, where it plays an important role in maintaining oxidative balance in the cell [152]. Studies have identified 237 mutations across coding and noncoding regions of the SOD1 gene in ALS (https://www.hgmd.cf.ac.uk, accessed on 17 January 2023), all of which affect SOD1 activity, albeit to different extents [153,154]. The deleterious effects of SOD1 mutations in ALS have been postulated to occur via a toxic gain of function, rather than by altered SOD1 activity [155] or an initial misfolding (with consequent gain of function within the cytosol and organelles), which results in impaired nuclear protection [154]. Mutant SOD1 could mediate these toxic effects either by generating cytotoxic amounts of H_2_O_2_ [156], reacting with nitric oxide to form large amounts of peroxynitrite [157] or forming toxic aggregates resulting from a decrease in the stability of SOD1 monomer/dimers [158]. Mutant SOD1 may also exhibit peroxidase activity and use the H_2_O_2_ generated during the enzyme reactions as a substrate to produce hydroxyl radicals [159]. Under conditions of oxidative stress, mutant SOD1 manifests several folding and aggregation defects that lead to the accumulation of toxic aggregates within neurons, exacerbating neuronal damage [160]. Pickles and colleagues [161] demonstrated that the accumulation of misfolded SOD1 in mitochondria within the spinal cord of SOD1G93A rats and SOD1G37R mice was associated with an increased susceptibility to oxidative stress and mitochondrial dysfunction. Furthermore, Ferri and colleagues [151] reported that oxidized cysteine residues in mutant SOD1 that were expressed in mouse motor neuronal NSC-34 cells mediated abnormal interactions with mitochondria, with a consequent shift in the mitochondrial redox balance, potentiating a higher level of oxidative stress. These studies show a consistent reciprocal relationship between the effects of mutant SOD1 and oxidative stress in propagating neuronal damage in ALS.

Other proteins involved in ALS pathophysiology have also been shown to display their effects through oxidative stress. Loss-of-function mutations in the gene for FUS have been shown to result in DNA strand breaks, which cause an increased vulnerability to oxidative stress, suggesting normal functioning of the protein is protective against oxidative stress in ALS [162]. C9orf72 mutations are the most prevalent mutations in ALS, with hexanucleotide expansion repeats being the most common [163]. These abnormal proteins preferentially bind to mitochondrial ribosomal proteins, increase mitochondrial membrane potential and ROS production and consequently promote oxidative stress-induced damage in ALS [164].

### 4.4. Huntington’s Disease

Huntington’s disease (HD) is an autosomal dominant, fully penetrant neurodegenerative disorder caused by an inherited CAG (polyglutamine) repeat expansion in the huntingtin (*HTT*) gene on chromosome 4p16.3 resulting in the production of a mutant huntingtin (mHTT) protein [165]. The mutation causes the misfolding/aggregation of huntingtin, with neurotoxic consequences [166,167]. Intriguingly, CAG repeats up to 39 are tolerated within the huntingtin protein, but individuals with greater than 39 repeats develop the disease. The CAG segment in the *HTT* gene is repeated approximately 25 times in healthy individuals, 40–50 times in adult-onset HD and more than 60 times in juvenile HD [165,168]. The disease affects medium spiny neurons in the striatum that appear to be selectively vulnerable to mHTT and later progresses to involve the thalamus and cerebral cortex. Damage to medium spiny neurons in selective regions of the striatum affects the basal nuclear pathway differently, with the initial losses affecting the indirect pathway, resulting in hyperkinetic movements and subsequent losses affecting the direct pathway that lead to hypokinetic movements. HD is clinically characterized by personality and mood changes that are sometimes followed by a cognitive decline [169]. Involuntary choreiform movements, bradykinesia, dystonia, rigidity and dementia are features of the disease, with death occurring approximately 15–20 years from the time of disease onset [169].

A number of studies have shown that oxidative stress is an important contributor to the pathophysiology of HD [170,171,172]. Markers of oxidative stress including 8-OHDG [173], products of protein carbonylation [172], 3-Nitrotyrosine [174], 4-HNE and isoprostanes [175] have been shown to be elevated in HD.

Mutant huntingtin impairs cellular DNA repair machinery by preventing the actions of the DNA repair protein ku70, thus mediating unchecked DNA damage [176]. Additionally, mutant huntingtin increases the level of ROS in neurons [177]. It can be inferred that this combination of unchecked DNA damage with oxidative stress leads to neuronal loss in HD. mHTT has also been shown induce cytotoxicity through its actions via the kynurenine pathway, with the attendant production of quinolinic acid and ROS, both of which propagate oxidative stress, mitochondrial dysfunction and neuronal death [178].

Mitochondrial dysfunction is considered a key player in HD pathogenesis. Sorolla and colleagues [179] reported the carbonylation of mitochondrial proteins including Cytochrome *bc1* complex subunit 2 and creatine kinase (directly involved in the production of ATP), as well as those involved in the glycolytic pathway, including glyceraldehyde-3P-dehydrogenase, and pyruvate kinase in the HD striatum. Carbonylation is an irreversible posttranslational modification that leads to the loss of protein function. The inactivity of these proteins increases mitochondrial free radical generation and sets up a vicious cycle. A previous study has shown that the levels and activities of antioxidant enzymes such as peroxiredoxin 1, 2 and 6 (Prx 1, 2 and 6), glutathione peroxidases 1 and 6 (GPX1 and 6) and mitochondrial superoxide dismutase in the HD striatum and cortex were markedly elevated [172], presumably as a countermeasure to the prevalent oxidative stress. Other studies have similarly shown depleted activities of electron transport chain proteins in the HD striatum [180,181]. Oxidative stress-induced mitochondrial DNA damage also plays an important role in the pathogenesis of HD, and studies have reported that mitochondrial DNA levels are depleted in neurons with mutant huntingtin compared to wild-type cells [182,183]. Altered mitochondrial calcium homeostasis has also been proposed as an important pathway by which mitochondrial dysfunction (from oxidative stress) causes neuronal loss in HD [184]. Mitochondria participate actively in the maintenance of intracellular Ca^2+^ homeostasis [185]. Calcium leakage has been observed in striatal and cortical neurons from HD animal models [186], where it leads to the gating of the mitochondrial permeability transition pore (mPTP) within the inner mitochondrial membrane and a consequent reduction in ATP production [187,188], presumably due to alterations in the mitochondrial membrane potential required for the proper functioning of ATP synthase. This ultimately results in neuronal death.

The studies above are a limited selection of many that reflect a consistent reciprocal relationship between the effects of mutant HTT and oxidative stress in the propagation of neuronal damage in HD.

## 5. The Oxytosis/Ferroptosis Cell Death Pathway and Oxidative Stress in Neuro-Degenerative Conditions

Studies of glutamate-induced excitotoxic cell death in a retinal cell line revealed a calcium-dependent form of delayed programmed cell death that failed to show the typical morphological or biochemical features of the well-known apoptotic cell death pathway [189]. This newly discovered form of cell death was characterized by the depletion of intracellular glutathione (GSH) stores and was exacerbated by the introduction of a culture medium low in cystine composition (a precursor to the amino acid cysteine, one of three amino acids required for GSH synthesis) with attendant increased oxidative stress and was inhibited by lipophilic antioxidants [190]. These findings represented the first evidence of the existence of the oxytosis/ferroptosis pathways of cell death. Glutamate exposure was shown to cause GSH depletion through the inhibition of cellular cystine uptake [189]. It was subsequently revealed that the transport system responsible for this cystine uptake was strikingly similar to System x_c_^−^, a cystine–glutamate antiporter [191]. An increase in cellular ROS with subsequent calcium influx was observed as a characteristic feature of this type of regulated cell death, and the term “oxytosis” was coined to reflect its trademark ROS accumulation [192]. In an attempt to discover novel compounds that are capable of inhibiting DNA synthesis in tumorigenic cells, Dolma and colleagues [193] discovered erastin, a compound that induced nonapoptotic cell death only in cells expressing the oncogenic allele of *HRAS* (*RASV^12^*) and Simian Virus 40 small T (ST) oncoproteins. Subsequent research revealed that this nonapoptotic cell death was characterized by mitochondrial dysfunction and ROS generation that was mitigated by lipophilic antioxidants [194]. In another study investigating small molecules with antiproliferative properties in the presence of the oncoprotein RAS, Yang and Stockwell [195] discovered the compound RSL3, which was reported to induce cell death in a similar manner, with characteristically elevated levels of intracellular iron, and the term “ferroptosis” was coined [196]. In particular, RSL3 was observed to inhibit glutathione peroxidase 4 (GPx4), which requires GSH for its activity, and it was suggested that GSH depletion and GPx4 inhibition work within the same pathway to promote cell death [197]. Erastin has subsequently been found to be a System x_c_^−^ inhibitor [198], and it is, thus, reasonable to assume that oxytosis and ferroptosis represent very similar (or perhaps the same) forms of regulated cell death.

During oxytosis, ROS, including superoxide and hydrogen peroxide, appear to be generated from complex I of the mitochondrial ETC via reverse electron transport from complex II [199,200,201]. As antimycin-A, a mitochondrial complex III inhibitor, has been shown to suppress erastin-induced cell death, it was inferred that mitochondrial ROS production is essential for erastin-induced ferroptosis [194]. As noted earlier, oxytosis/ferroptosis is characterized by the depletion of intracellular GSH stores, and GSH depletion has been reported in neurodegenerative diseases including AD [202] and PD [203], with the extent of GSH depletion correlating with disease severity. Other markers of active oxytosis/ferroptosis, including elevated lipoxygenase (LOX), lipid peroxides and calcium dysregulation, have also been described in various neurodegenerative conditions, including AD, PD, HD and ALS [204].

Compounds found to alleviate the effects of oxytosis/ferroptosis include Fisetin (3,3′,4′,7-tetrahydroxyflavone), a bioactive flavonol molecule found in many fruits and vegetables, which has the ability to maintain GSH levels in states of severe oxidative stress. The compound has been shown to mitigate the actions of 5LOX and 12/15LOX and prevent learning and memory deficits in APPswe/PS1dE9 transgenic AD [205] and senescence-accelerated SAMP8 mice [206]. The compound has shown beneficial effects in numerous other neurological conditions as well, and has been reviewed by Hassan and colleagues [207]. CNB001, a pyrazole derivative of curcumin, has also been shown to have significant inhibitory effects against oxytosis [208], and was later shown to reduce disease pathology in mouse models of AD [209] and PD [210]. The oxytosis/ferroptosis pathway is, therefore, an attractive candidate as a potential novel therapeutic target for neurodegenerative conditions.

## 6. Antioxidants and Antioxidant-Based Therapeutic Strategies for Neurodegenerative Diseases

We have established the important roles that oxidative stress plays in the pathophysiology of neurodegenerative disease. Antioxidant substances can therefore be presumed to be beneficial in the prevention and treatment of these diseases, especially as it has been shown that they are capable of reducing markers of oxidative stress when administered to both patients and animal models of the various diseases.

Cells employ various enzymatic and nonenzymatic antioxidant defense mechanisms to maintain pro-/antioxidant balance in order to mitigate the harmful effects of free radicals [211] (Table 3). Essential components of the enzymatic antioxidant defense system include superoxide dismutase (SOD), an enzyme that scavenges superoxide ions and converts them to molecular oxygen and hydrogen peroxide [162]; catalase (CAT), which decomposes hydrogen peroxide [212]; glutathione peroxidase (GPx), which reduces hydrogen peroxide to water and oxygen [213]; glutathione reductase (GR), which is primarily responsible for maintaining adequate levels of reduced glutathione [214] and thioredoxin reductase (TrxR, TR), an enzyme that exerts actions similar to glutathione reductase [215]. The glutathione redox cycle plays an important role in reducing potentially harmful intracellular peroxides. Glutathione peroxidase is a selenoprotein that contains four atoms of selenium in a tetrameric structure that forms the catalytic scaffold and requires glutathione as a co-substrate for its activity [216]. GPx is a cytosolic enzyme responsible for reducing lipid and hydroperoxides, thereby maintaining cell/neuronal membrane integrity, structure and overall cell function. Numerous studies investigating the therapeutic potential of antioxidant substances in neurodegenerative disease have been shown to act by upregulating GPx levels (in addition to other mechanisms), further supporting the roles of GPx as a pivotal antioxidant enzyme. A few examples of the antioxidant compounds employed to regulate GPx in vivo include a tomato seed extract that protected against neurotoxicity in a PD mouse model [217], imperatorin, a naturally occurring furanocoumarin that was shown to improve memory in an AD mouse model [218], WN1316, a novel acylaminoimidazole derivative that slowed disease progression in a late-stage ALS mouse model [219] and the dietary flavonoid rutin that was used in a 3-nitropropionicacid-induced, HD-like model in Wistar rats [220].

The nonenzymatic antioxidant system consists of substances including glutathione (GSH), vitamins A, E and C, thioredoxin (Trx), flavonoids, proteins (e.g., albumin, ceruloplasmin and metallothionein) and trace elements that exert direct influence in sequestering and reducing the deleterious effects of ROS and RNS [12].

In the following sections, we provide a very brief description of a few antioxidant substances that are being investigated as potential treatments for the neurodegenerative diseases discussed above. These sections are not intended to be an exhaustive review of the literature, and as such, a very limited number of studies are used to communicate the effects of antioxidants.

### 6.1. Alzheimer’s Disease

Currently approved therapies for Alzheimer’s disease are those designed to inhibit the action of acetylcholinesterase to improve acetylcholine levels in the brain and improve cognitive function (Donepezil, Rivastigmine and Galantamine), those designed to prevent glutamate-induced excitotoxicity (memantine) and the recently developed monoclonal antibody, Aducanumab, which mops up excess Aβ to reduce the accumulation of neuritic plaques in the brain. Oxidative stress plays a vital role in AD pathogenesis, and as such, antioxidant therapy has been widely studied and has been shown to display beneficial effects in the prevention and treatment of AD [221].

Vitamin E has been shown to have remarkable activity against peroxyl radicals [222], and can slow down their neurotoxic effects. Dong et al. [223] showed that AD patients had grossly reduced plasma vitamin E levels. Vitamin E significantly reduces oxidative and nitrosative damage in AD [224], and numerous clinical trials are ongoing to determine if vitamin E may offer significant therapeutic benefits in AD. Together with extracts of ginkgo biloba, vitamin E has been shown to improve cognitive function [225]. The actions of vitamin E also suppress tau-induced neurotoxicity in animal models [226]. The antioxidant actions of glutathione and its associated enzymes within the nervous system have been discussed. Mitochondrial glutathione depletion has been associated with increased Aβ-induced mitochondrial oxidative stress [227]. The administration of glutathione to a mouse model of AD has been shown to prevent neuroinflammation and rescue neurons from death [228]. Melatonin, a hormone synthesized in the pineal gland and associated with the circadian rhythm, scavenges oxygen and nitrogen-based free radicals and enhances the expression and activity of the antioxidant enzymes SOD and GPx [229], thereby reducing oxidative stress. It has also been reported that the antioxidant effects of melatonin can reduce the hyperphosphorylation of tau [230], thereby decreasing the propensity of neurofibrillary tangle formation, and can also inhibit Aβ-induced neurotoxicity [231]. The actions of metal ions in propagating oxidative damage have been discussed. Curcumin, a pigment compound found in turmeric that has been shown to possess powerful antioxidant properties, has been shown to prevent Aβ aggregation and oxidative stress [232]. Curcumin also replenishes glutathione levels in brain tissue in animal models of AD [233].

Other important antioxidant agents being evaluated as possible AD treatment options include ascorbyl palmitate [234], coenzyme Q [235], lipoic acid [236], resveratrol [237], silibinin [238] and gintonin [239]. Most of these substances are still being actively studied to determine any clinically significant effects in AD patients. Dietary sources of antioxidants have also been shown to have some beneficial effects in AD. Apple cider has been shown to increase the activities of SOD, CAT and GPx with resultant reduction in lipid peroxidation [240]. Diets rich in vitamin C, E, carotenoids, avonoids and polyphenols have been shown to complement conventional treatments in AD [241].

### 6.2. Parkinson’s Disease

As has been discussed, oxidative stress and mitochondrial dysfunction play a central role in the pathophysiology of Parkinson’s disease, and antioxidant molecules have been considered potential therapeutic options for PD.

A pilot study investigating the use of a high-dose combination therapy of vitamin C (3000 mg/day) and E (3200 IU/day) revealed that these antioxidants may slow PD progression [242]. A recent observational study supports this earlier finding. Hantikainen and colleagues [243] studied the diets of a large Swedish cohort (43,865) for a mean follow-up period of 17.6 years and discovered that dietary vitamin C and E intake was inversely correlated with the risk of PD. Participants in the highest tertile of dietary vitamin E consumption had a 32% lower risk of developing PD compared to participants in the lowest tertile [243]. A similar protective effect was discovered for vitamin C, as individuals who were in the highest tertiles of both vitamin E and C intake had a 38% lower risk of PD compared to participants in the lowest tertile [243]. Interestingly, a large cohort study reported that an increased dietary intake of vitamin E diminishes the risk of PD among both males and females, but vitamin E supplementation did not yield similar results [244]. There have been conflicting results, however, as a number of other studies have reported that vitamin E treatment failed to alter the clinical features [245] and life expectancy of PD patients [246]. More large-scale trials are warranted in order to establish the efficacy of vitamin E in PD treatment.

Coenzyme Q10 (CoQ10), also known as ubiquinone, is an endogenous antioxidant that acts as a cofactor in the electron transport chain during the production of ATP. As such, tissues with high rates of metabolism such as the brain, heart, liver and kidney require high levels of this antioxidant. Numerous studies have reported beneficial effects of CoQ10 in PD. A multicenter, randomized, parallel-group, placebo-controlled, double-blind, dosage-ranging trial of CoQ10 investigated the effects of 300, 600 or 1200 mg of CoQ10 per day, compared to placebo in 80 subjects with similar levels of disability who had not received any other treatment for 60 days prior to the commencement of the trial [247]. Results revealed a dose-dependent improvement in clinical features and activities of daily living, with subjects who received 1200 mg/day CoQ10 displaying a statistically significant improvement [247]. However, a later study by Beal and colleagues [248], who compared 1200 vs. 2400 mg/day CoQ10, subjects who also received 1200 IU/day of vitamin E showed no evidence of clinical benefit from high dosages of the antioxidant [248]. Clinical trials to determine the efficacy of CoQ10 are ongoing.

The important contributions of iron to mitochondrial dysfunction and oxidative stress in PD pathophysiology have been discussed above. Iron chelators are currently being investigated for their potential in limiting iron-induced oxidative stress in PD. Desferrioxamine has been shown to inhibit iron accumulation, with a resultant increase in GSSG, GSSG/GSH ratios and reduction in lipid peroxidation and levels of hydroxyl radicals [249]. 6-hydroxydopamine (6-OHDA) is known to induce oxidative stress in models of PD, partly through metal-catalyzed free radical formation. Intraventricular injections of Desferrioxamine and Deferasirox have been shown to prevent the depletion of dopamine levels in the striatum in a 6-OHDA rat model of PD [250].

In an MPTP-induced mouse model of PD, the administration of melatonin was found to prevent damage to the nigrostriatal pathway resulting from oxidative stress, neurotoxicity and neuronal degeneration caused by the toxin [251,252], with similar results shown in a 6-OHDA animal model of PD [253]. Melatonin also attenuated rotenone-induced glutathione depletion in the nigrostriatal pathway of PD mice and increased the activity of SOD and CAT in treated mice [254]. However, contradictory results have also been reported [255]. The antioxidant effects of creatine have also been shown to be potentially useful in PD treatment [256], although a recent meta-analysis of five RCTs that included 1339 participants called for more correlative studies as there was no observable clinical benefit in PD patients [257].

### 6.3. Huntington Disease

In the same vein, many of these antioxidant substances have been shown to hold potentially therapeutic effects in HD. Creatine, melatonin, vitamins C and E, CoQ10 and naturally occurring components such as lycopene and grape seed extracts in food products have all been shown to have potent antioxidant effects that may slow the course of the disease [258]. Novel synthetic compounds have also been investigated for possible therapeutic potential in HD. BN82451 is a brain-penetrable compound with neuroprotective effects, which it exerts by protecting the mitochondria against oxidative damage [259]. It has been shown in experimental studies to significantly prolong survival, improve motor function and gross morphology, striatal volume and striatal neuronal areas, and a cause significant decrease in the number of ubiquitinated cellular protein aggregates [259]. Another novel antioxidant compound with mitochondria-specific actions is XJB-5-131, which has been shown to significantly suppress oxidative damage to mitochondrial DNA and slow the pathophysiologic process in animal models of HD [260,261]. The dietary flavonoids rutin, myricetin and hesperidin have beneficial effects in the management of Huntington’s disease [262,263,264], typically by upregulating the neuroprotective and cytoprotective Nrf2 pathways. Nrf2 (nuclear erythroid 2-related factor 2) is a transcription factor that binds to the antioxidant response elements (ARE), regulating the expression of many genes involved in maintaining cellular oxidative balance [265].

### 6.4. Amyotrophic Lateral Sclerosis

Multiple studies have also demonstrated the efficacy of antioxidant substances in the management of ALS. Higher plasma vitamin E levels via supplementation has been shown to be somewhat protective against ALS onset [266,267]. Patients on combined riluzole and alpha-tocopherol (vitamin E) therapy had predominantly mild disease courses, with elevated glutathione and reduced TBARs (thiobarbituric acid reactive species—a by-product of lipid peroxidation) [268]. The administration of riluzole has been shown to possess antioxidant properties, mediated by the inhibition of protein kinase C [269], with an increase in glutathione synthesis, which occurs by the increase in intracellular levels of glutamate, a glutathione precursor [270].

*N*-acetyl-_L_-cysteine (NAC) is an antioxidant that alleviates free radical damage and replenishes plasma levels of cysteine, a glutathione precursor. In vitro studies have shown that NAC lowers mitochondrial ROS production in G93A SOD1-transfected SH-SY5Y human neuroblastoma cells [271]. An in vivo evaluation of NAC in the SOD1G93A mouse model of ALS by Andreassen and colleagues [272] revealed that oral treatment with a 1% solution in drinking water at 4–5 weeks of age significantly improved motor symptoms and extended survival in treated mice. A recent study investigating the efficacy of the intranasal administration of NAC tethered to a cell-penetrable nanocarrier showed significantly prolonged median survival time of SOD1 G93A transgenic mice [273]. A randomized, double-blind, placebo-controlled trial of NAC, however, did not result in a major increase in the 12-month survival or reduce disease progression in patients [274]. Intriguingly, patients with limb onset but not a bulbar onset of ALS did show a decrease in the one-year mortality rate by ~50% [274]. We have been unable to identify any other clinical trials of NAC, which may reflect its bioavailability. Studies have questioned the blood–brain barrier-penetrable potential of NAC, which is still in dispute.

CoQ10 exerts beneficial effects in ALS by scavenging free radicals and thereby protecting neurons and other tissues against oxidative stress. The administration of CoQ10 (200 mg/kg/day) to SOD1G93A-transgenic mice significantly prolonged the survival of treated mice when the administration commenced 50 days following birth [275]. A human case study reported substantially improved symptoms of grip strength, muscle wasting and mood following the consumption of 500 mg twice daily, followed by a dose of 200 mg twice daily after symptoms showed improvement [276]. However, a study that used 800 mg/kg/day of CoQ10 orally failed to prolong the survival of SOD1G93A mice when administered from the onset of disease [277]. A possible explanation for this contradictory result is the form of CoQ10 that was administered. CoQ exists in three redox states: a fully oxidized form commonly known as ubiquinone, a fully reduced form referred to as ubiquinol and an intermediate semiquinone radical (CoQ^•−^). Rationalizing that the reduced form ubiquinol, which is generally characterized by potent antioxidant properties, was difficult to use as it is readily oxidized in air, the authors compared 800 mg/kg ubiquinone_10_ to ubiquinol_10_ [277]. Although plasma levels of CoQ10 were significantly increased by both treatments, no effect on the disease progression and survival of SOD1G93A mice was found [277]. It is unclear why such a large dose of CoQ10 was used in this experiment, as it represents a 56-fold larger dose to that used by Kawasaki [276] and a 46-fold larger dose compared to that used to treat patients with PD [247].

The administration of melatonin to presymptomatic SOD1G93A-transgenic mice has been shown to delay disease progression and extended survival [278]. Zhang et al. [279] showed similar results, attributed to the inhibition of the caspase-1/cytochrome c/caspase-3 pathways. Dardiotis et al. [280], however, obtained completely opposite results, with an increase in motor neuron loss and 4-HNE levels, and an upregulation of toxic SOD1 expression. NADPH Oxidase (NOX) is an important regulator of ROS production in the CNS [281]. NOX inhibition in animal models of ALS slows disease progression and enhances survival [282]. Apocynin, a NOX inhibitor, has been shown to reduce O_2_^•−^ levels in human glioblastoma cells expressing mutant SOD and improve symptoms and significantly increase the lifespan of treated mice [283]. Later studies, however, were unable to replicate these results [284]. The NAD^+^/SIRT1 pathways are involved in mitochondrial metabolism and the maintenance of oxidative balance [285], with reduced sirtuin (SIRT1) levels discovered in ALS patients [286]. The administration of resveratrol, a SIRT1 activator in mice models of ALS, was found to protect against mitochondrial damage and improve symptoms [287,288].

A novel small molecular acylaminoimidazole derivative, WN1316, that selectively suppresses oxidative stress-induced cell death and neuronal inflammation by upregulating Nrf2 and increasing GSH, thus offering protection against oxidative stress, was shown to improve motor function and extend the survival of SOD1^H46R^- and SOD1^G93A^-transgenic mice [219]. A phase I clinical trial to determine the tolerability of WN1316 was completed in 2015, but results have not been published (UMIN000015054).

Results from the section above are summarized in Table 4.

Results presented in this section support the notion that antioxidants are powerful entities for the treatment of oxidative stress. However, what is clear from the literature is that antioxidants are not a treatment for neurodegenerative diseases. As most neurodegenerative diseases develop as a consequence of interactions between genes and the environment, a combination of antioxidants with other available therapeutic options must be considered in a multipronged approach to treat these devastating diseases of the nervous system.

## 7. Conclusions

The imbalance between pro-oxidant/free radical levels and antioxidant defense systems appears to be a universal condition in neurodegeneration, with the system often skewed towards excess pro-oxidants. Generally, it has been well established that this state of oxidative stress plays important roles in propagating many pathophysiologic processes involved in the progression of most neurodegenerative conditions. Many preclinical and clinical studies have consistently demonstrated the presence of many signs and markers of oxidative stress in both animal models and humans with various neurodegenerative diseases. However, these studies have also consistently shown that the relationship between oxidative stress and these diseases is complex and very often reciprocal, with oxidative stress promoting the development of the classic pathologic features of these diseases and vice versa. Furthermore, studies have shown that different neurodegenerative conditions often express many oxidative stress biomarkers in a diverse manner and in varying levels, thus making the identification of a potentially uniform oxidative stress biomarker for specific neurodegenerative diseases challenging. This may also make the assessment of the efficacy of proposed antioxidant-based therapies for neurodegenerative diseases challenging. Many antioxidant-based therapeutic strategies have been studied, with many ongoing, and they have also shown quite inconsistent results, which are often difficult to adopt. This extensive heterogeneity may be explained by the differences in the nature of preclinical and clinical studies. The fact that most of the clinical studies have been among small study populations may also explain the heterogeneity and make the adoption of findings from those studies difficult. Both natural and novel synthetic therapeutic agents are being studied for their antioxidant benefits and clinical effects in neurodegenerative disease states. More extensive, large-scale preclinical and clinical studies of oxidative stress biomarkers and therapies are required in order to obtain more consistent and clinically adoptable results, which would go a long way in ultimately improving the quality of life of patients suffering from neurodegenerative disease conditions.

## Figures and Tables

**Table 1:** Key reactive oxygen species, the reactions that generate them and their physiological and pathological properties.

Reactive Oxygen Species		
Species	Biochemical Pathway	Roles
Superoxide (O_2_^•−^)	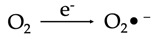	Physiological Involved in innate immune system function—oxygen-dependent destruction of invading pathogens Signaling in vasodilation, chemotaxis, leukocyte adhesion and pain Regulates gene expression, aging, senescence and apoptosis General mediation of inflammatory processes and Toll-Like Receptor (TLR) pathways Regulation of cellular differentiation Pathological Produces peroxynitrite Oxidizes biomolecules such as lipids, proteins and DNA Damages cell membranes, alters signal transduction mechanisms and impairs DNA repair capabilities
Hydroxyl radical (HO^•^)	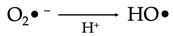	Physiological Regulation of adaptation to hypoxia Regulation of autophagy Regulation of cellular differentiation Regulation of TLR pathways and inflammation Pathological Oxidizes biomolecules such as lipids, proteins and DNA Damages cell membranes, alters signal transduction mechanisms and impairs DNA repair abilities
Hydrogen peroxide (H_2_O_2_)	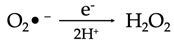	Physiological Posttranslational protein modifications Regulation of signaling pathways Regulation of specific enzyme activities, e.g., phosphatases Regulation of cellular differentiation Pathological Oxidizes biomolecules such as lipids, proteins and DNA Damages cell membranes, alters signal transduction mechanisms and impairs DNA repair abilities

**Table 2:** Key reactive nitrogen species, the reactions that generate them and their physiological and pathological properties.

Reactive Nitrogen Species		
Species	Biochemical Pathway	Roles
Nitric oxide (NO)	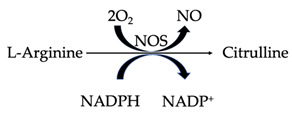	Physiological Controls vascular tone Involved in vascular permeability Important for cell adhesion Important for platelet adhesion Neurotransmitter Pathological Nitrosylation of proteins Inhibits platelet aggregation Activates p21^ras^ and plays a role in proliferation and differentiation Triggers apoptosis Produces ROS and is precursor to peroxynitrate
Peroxynitrite (ONOO^−^)	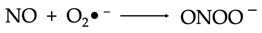	Physiological Contributes to host–defense response and inflammation Activates stress response pathways such as MAPK Enhances myocardial contractility and exerts cardioprotective effects Pathological Oxidizing agent Produces nitrite ions Lipid peroxidation Oxidative modification of bases in nucleotides Mitochondrial toxicity Cell death

**Table 3:** Enzymatic and nonenzymatic antioxidant systems.

Antioxidant Agent	Mechanism of Action
*Enzymatic*	
Superoxide Dismutases (SOD)	Scavenges superoxide ions to convert them to hydrogen peroxide
Catalases (CAT)	Breaks down hydrogen peroxide
Peroxiredoxins (Prx)	Reduces hydrogen peroxide to water
Glutathione peroxidase (GPx)	Reduces hydrogen peroxide to water
Glutathione reductase (GR)	Converts oxidized glutathione (GSSG) to reduced glutathione (GSH)
Glutathione S Transferase	Conjugates harmful xenobiotics to glutathione
Thioredoxin reductase	Repairs oxidative damages to proteins
*Nonenzymatic*	
Vitamin A	ROS scavenger
Vitamin C	ROS scavenger
Vitamin E	ROS scavenger
Melatonin	ROS scavenger
Lycopene	ROS scavenger
α-Lipoic acid	ROS scavenger
Polyphenols—Phenolic acid and flavonoids	ROS scavenger, metal chelation and induction of antioxidant enzymes

**Table 4:** Effects of antioxidant therapies in neurodegenerative diseases.

Antioxidant	Biomarker Changes	Survival	ALS	HD	AD	PD
Vitamin E	Increased GPx, reduced 8-OHG and TBARs	Increased	Desnuelle et al., 2001 [268]	-	-	-
Edaravone	Reduced 3NT in CSF	Increased	Yoshino and Kimura, 2006 [289]	-	-	-
Melatonin	Reduced protein carbonyls in serum	Increased	Weishaupt et al., 2006 [278]	-	-	-
Melatonin	Increased 4HNE and SOD	Reduced	Dardiotis et al., 2013 [280]	-	-	-
Melatonin	Increased SOD, GPx and CAT	Increased	-	-	-	Kaya et al., 2013 [290]
*N*-Acetyl Cysteine	Reduced SOD, increased GPx and GSH	Increased	-	-	-	Sharma et al., 2007 [291]
*N*-Acetyl Cysteine	Increased GSH	Increased	Andreassen et al., 2000 [272]	-	-	-
Deferrioxamine	Increased GSH and SOD	-	-	-	-	Lan and Jiang, 1997 [249]; Dexter et al., 2011 [250]
Vitamin E+, Vitamin C+, Lipoic acid and CoQ10	Reduced F2 isoprostanes	-	-	-	Galasko et al., 2012 [292]	-
Curcumin	No change in F2 isoprostanes, increased GSH	Increased	-	-	Nishinaka et al., 2007 [233]	-
Melatonin	Reduced protein carbonyls and SOD	Increased	-	Khan et al., 2018 [258]	-	-
